# Relationship between Body Roundness Index and Risk of Type 2 Diabetes in Japanese Men and Women: A Reanalysis of a Cohort Study

**DOI:** 10.1155/2021/4535983

**Published:** 2021-12-29

**Authors:** Wei Zhao, Jingjing Tong, Jinghua Li, Yongtong Cao

**Affiliations:** ^1^Department of Clinical Laboratory, China-Japan Friendship Hospital, Beijing 100029, China; ^2^Liver Failure Treatment and Research Center, The Fifth Medical Center of PLA General Hospital, Beijing 100039, China

## Abstract

**Purpose:**

The purpose of this study was to investigate the association between body roundness index (BRI) and type 2 diabetes (T2DM) in each sex, explore the dose-response relationship between them, and evaluate the predictive value of BRI for T2DM.

**Materials and Methods:**

A retrospective cohort study was performed on 15,464 Japanese patients at the Murakami Memorial Hospital. Data on anthropometric indices and biochemical parameters were obtained. Multivariate Cox regression models were used to estimate the hazard ratios (HRs) of incident T2DM associated with BRI. Dose-response relationships were evaluated using a smoothing function analysis and the threshold effect. Receiver operating characteristic curves were used to evaluate and compare the predictive values of BRI, body mass index (BMI), and waist circumference (WC) for T2DM.

**Results:**

During a median 5.4-year follow-up period, 373 subjects were diagnosed with T2DM. After adjusting for age, alcohol intake, smoking status, fatty liver, systolic blood pressure, fasting plasma glucose, glycated hemoglobin, high-density lipoprotein cholesterol, triglycerides, and total cholesterol, the relationship between BRI and T2DM was linear in women (HR (95% CI) for BRI *Z* score = 1.48 (1.26,1.74)) and curvilinear in men (HR (95% CI) on the left and right of the inflection point = 0.70 (0.44, 1.10) and 1.46 (1.27, 1.67), respectively). Compared with BMI (area under the curve (AUC) = 0.684; *p* < 0.001) and WC (AUC = 0.700; *p*=0.007), BRI was the strongest predictor of T2DM in men (AUC = 0.715). Similarly, the AUC of BRI was larger than that of BMI (AUC = 0.757; *p*=0.966) and WC (AUC = 0.733; *p*=0.015) in women.

**Conclusions:**

BRI was positively linearly associated with an elevated risk of incident T2DM in women. In men, the relationship between BRI and T2DM was J-shaped. BRI is an effective indicator of predicting T2DM. Its discriminatory power was higher than that of BMI and WC in both sexes.

## 1. Introduction

In 2019, the International Diabetes Federation reported that 463 million people aged 20–79 years were diagnosed with diabetes mellitus, and type 2 diabetes mellitus (T2DM) was the most common [1]. T2DM can be prevented through physical activity and a low-fat diet aimed at weight reduction [2]. Therefore, identifying people at high risk of T2DM is essential and may help prevent an unprecedented increase in the incidence of the disease.

Obesity is a known risk factor for T2DM [3]. Body mass index (BMI) is the most commonly used indicator of obesity [4, 5]. The relationship between BMI and T2DM has already been described by several studies [6, 7]. Though BMI can be easily determined, it fails to reflect body fat distribution [8, 9]. Reportedly, the abdominal visceral adipose tissue is more strongly associated with obesity-related comorbidities than the peripheral adipose tissue [7, 10, 11].

The body roundness index (BRI), which was first proposed by Thomas et al. in 2013, can predict the proportions of body fat and visceral adipose tissue in individuals [12]. Several cross-sectional studies have suggested a positive association between BRI and T2DM [13, 14]. BRI is an independent risk factor for and a predictor of T2DM incidence [15, 16]. However, the association between BRI and T2DM in both sexes has not been reported. Furthermore, to the best of our knowledge, the dose-response relationship between BRI and T2DM has not been elaborated to date. It remains controversial whether BRI is a better anthropometric indicator than BMI for predicting the occurrence of diabetes [13, 14, 16].

In the present study, we aimed to evaluate the association between BRI and T2DM in Japanese men and women, explore the dose-response relationship, and investigate whether BRI has a superior predictive value for T2DM than traditional anthropometric indices such as BMI and waist circumference (WC).

## 2. Materials and Methods

### 2.1. Data Source

The data reported in this study have been deposited in the public Dryad Digital Repository (https://datadryad.org/stash/dataset/doi:10.5061/dryad.8q0p192). This database is a curated resource that makes research data discoverable, freely reusable, and citable. We have previously cited the Dryad data set [17, 18] based on the Dryad Terms of Service.

### 2.2. Study Design and Participants

In this study, we reanalyzed the NAfld in the Gifu Area, Longitudinal Analysis (NAGALA) study conducted in Japan. We have described the details of this study previously [19, 20]. This was a retrospective cohort study of a medical examination program at the Murakami Memorial Hospital (Gifu, Japan) conducted from 2004 to 2015. A total of 20,944 participants aged 18–79 years who completed at least two examinations of the program were recruited and screened based on the following exclusion criteria: (1) alcoholic fatty liver disease, (2) viral hepatitis (including HBV and HCV), (3) use of any medication at baseline, (4) impaired fasting glucose or T2DM at baseline examination, and (5) missing or incorrect covariate data. As stated previously, the ethics committee of the Murakami Memorial Hospital approved this study, and all participants signed informed consent forms [17].

### 2.3. Data Collection and Measurement

All participants completed a questionnaire that was used to obtain their medical history and lifestyle information, including alcohol and smoking habits and physical activity [17]. Anthropometric indices including height, weight, WC, systolic blood pressure (SBP), and diastolic blood pressure (DBP) were measured by experienced nurses. BMI and BRI were calculated using the following equation: BMI = weight (kg)/height (m)^2^ and BRI = 364.2–365.5 × {1–[(WC(m)/2*π*)/(0.5 × height(m))]^2^}^1/2^. Fatty liver was diagnosed only based on abdominal ultrasonography [17].

Blood samples were collected from participants after 8 h of fasting. Samples were centrifuged immediately and stored at −80°C until analysis. Fasting serum samples were used to determine levels of triglycerides (TG), total cholesterol (TC), high-density lipoprotein cholesterol (HDL-C), and fasting plasma glucose (FPG) using MODULAR ANALYTICS (Hitachi High-Technologies Corp. Ltd., Tokyo, Japan). Whole blood samples were used to assay glycated hemoglobin (HbA1c) using high-performance liquid chromatography.

### 2.4. Definition of T2DM

T2DM was defined as FPG ≥7.0 mmol/L and HbA1c ≥ 6.5% according to the American Diabetes Association criteria or self-reported clinician-diagnosed diabetes [21].

### 2.5. Statistical Analysis

Mean ± SD or median (Q1–Q3) was used for representing continuous variables, and number (percentage) was used for representing categorical variables. The baseline characteristic differences among quartiles of BRI were tested with one-way ANOVA (normal distribution), the Kruskal–Wallis H test (skewed distribution), and the *χ*^2^ test (categorical variables). Multivariate Cox regression models were used to study the effects of BRI on incident T2DM. Potential confounders were screened by altering the initial regression coefficients by at least 10% when added to the basic model. In this study, the screened confounders included age, alcohol intake, smoking status, fatty liver, SBP, FPG, HbA1c, HDL-C, TG, and TC. In addition, a generalized additive model was used to assess nonlinear relationships. The threshold effect was examined using a two-piecewise linear regression model according to the smoothing plot. A recurrence method was used to determine the BRI threshold level at which the relationship between BRI and T2DM began to change and became significant. The inflection point was moved along a predefined interval and was chosen when it yielded the maximum likelihood model. To evaluate the performance of BRI in predicting T2DM, receiver operating characteristic (ROC) curve analysis was performed, and the optimal cutoffs were obtained from the Youden index (sensitivity + specificity – 1). The areas under the ROC curves (AUC) were compared using a nonparametric test.

All statistical analyses were performed using R (The R Foundation, Vienna, Austria) and EmpowerStats (X&Y Solutions, Inc., Boston, MA, USA). Results with *p* < 0.05 (two-sided) were considered statistically significant.

## 3. Results

### 3.1. Selection of Participants

Of the 20,944 participants, 5,480 were excluded from this study. Of these excluded subjects, 739 had a heavy drinking habit; 416 had viral hepatitis; 2,321 used medications; 1,131 had pathologically high FPG levels or T2DM; and 873 had missing or incorrect data. The remaining 15,464 participants (7,034 women and 8,430 men) were included in the data analysis. Finally, 373 subjects were diagnosed with T2DM after a median 5.4-year follow-up period.

### 3.2. Baseline Characteristics according to BRI Quartiles

The baseline characteristics of the study participants according to the BRI quartiles are shown in Table 1. The mean age of all participants was 43.7 ± 8.9 years, and 45.49% were women. The mean BRI was 2.57 ± 0.93 for women and 2.88 ± 0.84 for men. Men and women with higher BRI tended to be older and physically inactive, and they were more likely to have fatty liver, greater WC, higher blood pressure, and higher BMI. Furthermore, BRI was directly proportional to TG, TC, FPG, and HbA1c levels and inversely proportional to HDL-C levels in both sexes. Baseline characteristics by sex are shown in Supplementary Table 1. HDL-C and HbA1c levels were lower in men than in women. In contrast, all other variables were significantly higher in men than in women (*p* < 0.001). Similar trends of all characteristics except age, physical activity, BRI, and TC levels were found in men and women with T2DM (Supplementary Table 1).

### 3.3. Association between BRI and T2DM

Cox regression models were used to evaluate the HRs and 95% CIs of incident T2DM associated with BRI.  Table 2 shows the nonadjusted and all-adjusted models per sex. In the nonadjusted models, BRI showed a positive association with the risk of T2DM in both sexes as the quartile of BRI increased. After adjustment for age, alcohol intake, smoking status, fatty liver, SBP, FPG, HbA1c, HDL-C, TG, and TC, a similar trend was observed only in women. Compared with BRI quartile 1, the HRs and 95% CIs were 1.59 (0.57, 4.43), 1.66 (0.62, 4.44), and 2.74 (1.05, 7.14) for BRI quartiles 2–4, respectively (*P* for trend = 0.019). In men, the relationship between BRI and T2DM decreased first and then increased after full adjustment. The HRs and 95% CIs were 0.64 (0.38, 1.07), 0.55 (0.33, 0.92), and 1.05 (0.64, 1.72) for BRI quartiles 2–4, respectively. As a continuous variable, BRI was always significantly associated with the risk of T2DM in both sexes, irrespective of adjustment. In the fully adjusted model, for every one-unit increase in the BRI *Z* score, the risk of T2DM increased by 48% in women (HR (95% CI) = 1.48 (1.26, 1.74)) and 34% in men (HR (95% CI) = 1.34 (1.18, 1.52]).

### 3.4. Threshold Effect Analysis of BRI for T2DM

**Figure 1:**
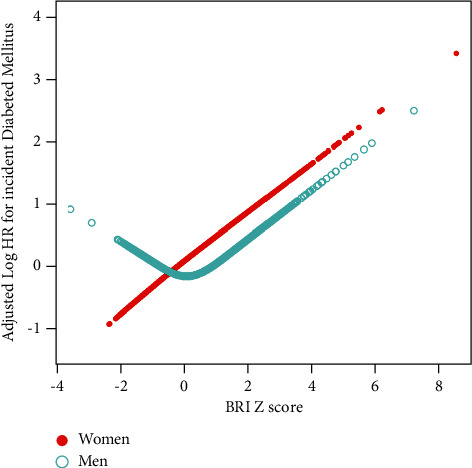
Dose-response relationship between BRI and incident T2DM by gender in the NAGALA study. The solid dots indicate the estimated risk of incident T2DM in women, and the hollow dots represent the estimated risk of incident T2DM in men adjusted for age, alcohol intake, smoking status, fatty liver, SBP, FPG, HbA1c, HDL-C, TG, and TC. BRI, body roundness index.

Because of the inconsistent risk of developing T2DM when BRI was used as a categorical or continuous variable, analyses of nonlinear relationships were necessary. In the fully adjusted model, the relationship between BRI and T2DM in women was always linear, while a nonlinear relationship was observed in men (Figure 1). Using a two-piecewise linear regression model, we calculated the inflection points, which were 2.85 for BRI. On the left of the inflection point, the risk of developing T2DM decreased as the BRI *Z* score increased (HR (95% CI) = 0.70 (0.44, 1.10); *p*=0.1233). However, the opposite relationship was observed on the right of the inflection point (HR (95% CI) = 1.46 (1.27, 1.67); *p* < 0.001) (Table 3).

### 3.5. Predictive Value of BRI for T2DM

**Figure 2:**
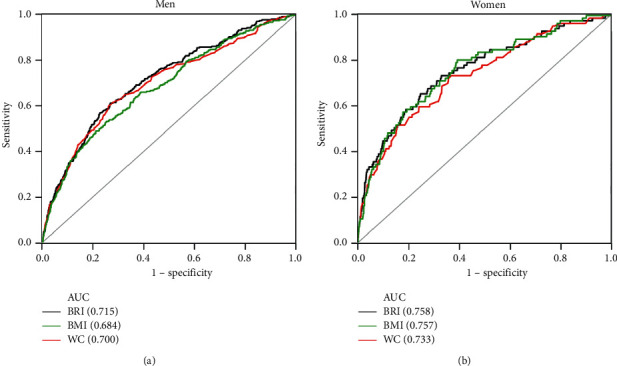
ROC curves for BRI, BMI, and WC in men (a) and women (b) in the NAGALA study. AUC of BRI, BMI, and WC to identify participants with T2DM according to sex. BRI, body roundness index; BMI, body mass index; WC, waist circumference (cm); AUC, area under the curve.

The results of the ROC analysis for BRI, BMI, and WC are shown in Figure 2 and Table 4. ROC analysis revealed that BRI was the strongest predictor of T2DM in men (AUC = 0.715) compared with BMI (AUC = 0.684; *p* < 0.001) and WC (AUC = 0.700; *p*=0.007). The AUC for BRI in women (AUC = 0.758) was significantly larger than that for WC (AUC = 0.733; *p*=0.015) but not for BMI (AUC = 0.757; *p*=0.966). The optimal cutoffs for BRI were 3.29 in men (sensitivity 61.2% and specificity 73.2%) and 3.05 in women (sensitivity 65.5% and specificity 75.3%).

## 4. Discussion

In this study, we found that BRI was positively correlated with an increased risk of T2DM in women. However, a curvilinear relationship between BRI and T2DM was observed in men. The predictive value of BRI for T2DM was superior to that of BMI and WC in both sexes.

Obesity rates are increasing rapidly worldwide, and this is associated with many health problems [22, 23]. BRI, which is a new index used to define obesity, can indicate abdominal deposition of adipose tissue [12]. The effect of BRI on metabolic syndrome has been studied sufficiently [24–26]. In recent years, the association between BRI and incident T2DM has been reported in several studies. These studies show that BRI was positively correlated with T2DM incidence [13–16]. To the best of our knowledge, only two cohort studies have assessed the association between BRI and T2DM. In a Chinese 15-year follow-up prospective study (*n* = 687), log_10_BRI (HR (95% CI): 2.16 (1.63, 2.88) per SD; *p* < 0.001) was significantly associated with incident diabetes after adjusting for potential confounding factors [15]. Another cohort study with 9,962 participants in China showed that the HRs (95% CI) for BRI were 1.60 (1.20, 2.13) in men and 1.73 (1.21, 2.46) in women [16]. Our findings on BRI are consistent with those of previous studies.

However, the type of relationship between BRI and incident T2DM has not been elaborated on previously. In this study, we observed a positive linear relationship between BRI and the risk of T2DM across the entire range of BRI values in women. In men, the relationship between BRI and T2DM was J-shaped. The participants whose BRI was approximately 2.85 had the lowest risk of developing T2DM. A nonlinear relationship between BMI and the risk of death was reported in 2008. The lowest risk of death was observed when BMI was 25.3 kg/m^2^ for men and 24.3 kg/m^2^ for women [27]. An analysis including 57 prospective studies indicated that mortality related to BMI was the lowest at approximately 22.5–25 kg/m^2^ in both sexes [28]. Similar relationships were also found between WC or waist-to-height ratio and all-cause mortality in both sexes [29]. All these studies indicate that a person who is extremely fat or thin has a higher mortality risk, and our findings on T2DM support this conclusion.

Interestingly, the association of BRI with T2DM showed different trends in each sex. Many sex-based differences have been reported in the development of T2DM [30]. First, sex steroid hormones protect women from T2DM [31]. Second, women have a greater capacity for insulin secretion and are less prone to insulin resistance than men [32]. In addition, there are some metabolic differences between men and women that affect the incidence of T2DM [33]. Besides, women are prone to store adipose tissue in subcutaneous sites, whereas men preferentially store adipose tissue in the visceral area due to the differences in fat metabolism and sex hormones [34–36]. These may partly explain the sex disparities in dose-response patterns, as BRI is an indicator of the amount of visceral adipose tissue. Our analysis also showed that the dose-response relationship between BRI and T2DM changed in men and women without fatty liver (data not shown), indicating that fatty liver might cause sex-based differences. In addition, HDL-C, TC, and TG levels might not play an important role in sex-based differences (data not shown). However, our database did not include the indices of sex hormones and insulin resistance, and we could not further analyze their roles in sex-based differences. Furthermore, the proportion of subjects who developed T2DM was slightly lower among women (87/7034), and larger cohorts are needed to confirm this phenomenon.

To date, there is no comprehensive consensus on the best anthropometric index to predict diabetes. Wang et al. found that BRI had a higher Harrell's C-statistic of 0.714 (0.658, 0.770) than WC at 0.701 (0.644, 0.759) [15]. A study in northeast China reported that BRI showed the highest AUC values (0.66 in men and 0.67 in women) for T2DM in both sexes compared with other anthropometric indices, including BMI (0.63 in men and 0.62 in women) and WC (0.65 in men and 0.66 in women) [13]. However, a prospective study on elderly Chinese subjects revealed that BMI was the strongest predictor of diabetes in both sexes compared with WC and BRI [16]. In this cohort study, the AUC of BRI was the largest compared with those of BMI and WC for both sexes, though the difference between the AUCs for BRI and BMI in women was not significant (*p*=0.966). The optimal cutoffs for BRI in this study were 3.29 in men (sensitivity 61.2% and specificity 73.2%) and 3.05 in women (sensitivity 65.5% and specificity 75.3%), similar to previous research [16].

Our study has several strengths. First, it was a large, population-based retrospective cohort study. Second, all previous studies were conducted in China. This is the first study focusing on Japanese subjects, and our findings on BRI are consistent with those earlier studies. Third, we screened for confounding factors using strict statistical methods [20]. Previously, some studies only adjusted for sex and age, and some did not adjust for biochemical indices. In this study, we adjusted for age, alcohol intake, smoking status, fatty liver, SBP, FPG, HbA1c, HDL-C, TG, and TC levels.

There are some limitations to our study. First, we did not distinguish type 1 diabetes from type 2 diabetes, though the incidence of type 1 diabetes is very low in Japan [37]. Therefore, the HRs of incident T2DM might have been overestimated. Second, the incidence of T2DM might have been underestimated in this study because oral glucose tolerance tests were not performed. Third, as all participants were Japanese, these findings may not be generalizable to non-Japanese populations.

## 5. Conclusions

BRI was associated with T2DM and was an effective predictor of the incidence of the disease. The discriminatory power of BRI was higher than that of BMI and WC.

## Figures and Tables

**Table 1:** Baseline characteristics of participants in men and women by categories of BRI in the NAGALA study.

	BRI quartiles				*p* value
	Quartile 1	Quartile 2	Quartile 3	Quartile 4	
Men	0.87–2.30	2.30–2.81	2.81–3.37	3.37–9.21	
Participants (*n)*	2108	2107	2107	2108	
Age (years)	41.4 ± 8.6	43.3 ± 8.6	45.3 ± 8.80	46.3 ± 9.2	<0.001
Current smokers	789 (37.46%)	745 (35.36%)	741 (35.17%)	751 (35.63%)	<0.001
Alcohol intake (g/wk)	18 (1–90)	36 (1–126)	36 (1–126)	25.7 (1–126)	<0.001
Fatty liver	63 (2.99%)	300 (14.24%)	683 (32.42%)	1209 (57.35%)	<0.001
Physical activity	439 (20.85%)	456 (21.64%)	380 (18.04%)	324 (15.37%)	<0.001
BMI (kg/m^2^)	18.0 ± 1.6	22.1 ± 1.4	23.7 ± 1.5	26.4 ± 2.6	<0.001
WC (cm)	71.5 ± 3.8	77.9 ± 2.9	82.6 ± 3.3	89.9 ± 5.8	<0.001
SBP (mmHg)	112 ± 12	117 ± 13	120 ± 13	126 ± 15	<0.001
DBP (mmHg)	70 ± 9	74 ± 9	76 ± 9	80 ± 10	<0.001
TC (mmol/L)	4.86 ± 0.80	5.12 ± 0.82	5.24 ± 0.83	5.42 ± 0.86	<0.001
HDL-C (mmol/L)	1.47 ± 0.38	1.34 ± 0.34	1.25 ± 0.32	1.16 ± 0.27	<0.001
TG (mmol/L)	0.57 (0.47–0.90)	0.86 (0.62–1.23)	1.04 (0.75–1.50)	1.25 (0.88–1.82)	<0.001
FPG (mmol/L)	5.18 ± 0.36	5.29 ± 0.36	5.35 ± 0.37	5.41 ± 0.35	<0.001
HbA1c (%)	5.1 ± 0.3	5.1 ± 0.3	5.2 ± 0.3	5.2 ± 0.3	<0.001

Women	0.63–1.91	1.91–2.40	2.40–3.06	3.06–10.39	
Participants (*n*)	1759	1758	1758	1759	
Age (years)	40.4 ± 8.4	41.7 ± 8.2	43.9 ± 8.3	47.0 ± 8.7	<0.001
Current smokers	127 (7.22%)	120 (6.83%)	105 (5.97%)	102 (5.80%)	0.031
Alcohol intake (g/wk)	1 (0–4.2)	1 (0–9.3)	1 (0–9.1)	1 (0–4.2)	0.106
Fatty liver	4 (0.23%)	17 (0.97%)	76 (4.32%)	389 (22.11%)	<0.001
Physical activity	303 (17.23%)	293 (16.67%)	267 (15.19%)	246 (13.99%)	0.036
BMI (kg/m^2^)	18.5 ± 1.5	19.9 ± 1.5	21.3 ± 1.7	24.3 ± 3.0	<0.001
WC (cm)	63.0 ± 3.2	68.4 ± 2.6	73.3 ± 2.9	82.1 ± 6.3	<0.001
SBP (mmHg)	104 ± 12	106 ± 12	110 ± 13	117 ± 15	<0.001
DBP (mmHg)	64 ± 8	66 ± 9	68 ± 10	72 ± 10	<0.001
TC (mmol/L)	4.89 ± 0.83	4.94 ± 0.82	5.12 ± 0.86	5.40 ± 0.90	<0.001
HDL-C (mmol/L)	1.76 ± 0.38	1.70 ± 0.36	1.64 ± 0.39	1.50 ± 0.35	<0.001
TG (mmol/L)	0.49 (0.36–0.67)	0.51 (0.37–0.71)	0.58 (0.41–0.84)	0.77 (0.53–1.10)	<0.001
FPG (mmol/L)	4.87 ± 0.37	4.92 ± 0.37	5.01 ± 0.38	5.15 ± 0.40	<0.001
HbA1c (%)	5.1 ± 0.3	5.1 ± 0.3	5.2 ± 0.3	5.3 ± 0.3	<0.001

*Note*. Data are presented as mean ± SD, median (Q1–Q3), or *N* (%). BRI, body roundness index; BMI, body mass index; WC, waist circumference; SBP, systolic blood pressure; DBP, diastolic blood pressure; TC, total cholesterol; HDL-C, HDL-cholesterol; TG, triglycerides; FPG, fasting plasma glucose; HbA1c, glycated hemoglobin.

**Table 2:** Association between BRI and incident T2DM by gender in the NAGALA study.

Exposure	Nonadjusted		Adjust I		Adjust II		Adjust III	
	HR (95% CI)	*p* value	HR (95% CI)	*p* value	HR (95% CI)	*p* value	HR (95% CI)	*p* value
Men								
BRI *Z* score	1.94 (1.78, 2.11)	<0.001	1.96 (1.78, 2.15)	<0.001	1.50 (1.34, 1.69)	<0.001	1.34 (1.18, 1.52)	<0.001

BRI quartile								
BRI Q1	1 (reference)		1 (reference)		1 (reference)		1 (reference)	
BRI Q2	1.40 (0.86, 2.30)	0.178	1.35 (0.83, 2.22)	0.228	1.04 (0.63, 1.71)	0.892	0.64 (0.38, 1.07)	0.086
BRI Q3	1.99 (1.26, 3.15)	0.003	1.80 (1.14, 2.86)	0.012	0.99 (0.61, 1.62)	0.968	0.55 (0.33, 0.92)	0.023
BRI Q4	6.09 (4.05, 9.14)	<0.001	5.35 (3.55, 8.07)	<0.001	2.13 (1.33, 3.40)	0.002	1.05 (0.64, 1.72)	0.841
*P* For trend	<0.001		<0.001		<0.001		0.009	

Women								
BRI *Z* score	2.12 (1.92, 2.35)	<0.001	2.11 (1.88, 2.35)	<0.001	1.68 (1.44, 1.97)	<0.001	1.48 (1.26, 1.74)	<0.001

BRI quartile								
BRI Q1	1 (reference)		1 (reference)		1 (reference)		1 (reference)	
BRI Q2	2.11 (0.76, 5.81)	0.15	1.96 (0.71, 5.42)	0.193	1.89 (0.68, 5.23)	0.22	1.59 (0.57, 4.43)	0.375
BRI Q3	3.59 (1.39, 9.29)	0.008	3.16 (1.22, 8.19)	0.018	2.32 (0.88, 6.11)	0.087	1.66 (0.62, 4.44)	0.313
BRI Q4	14.4 (6.17, 33.7)	<0.001	11.3 (4.77, 26.61)	<0.001	5.18 (2.05, 13.08)	<0.001	2.74 (1.05, 7.14)	0.039
*P* For trend	<0.001		<0.001		<0.001		0.019	

*Note*. Adjust I model adjusts for age; Adjust II model adjusts for adjust I + alcohol intake, smoking status, fatty liver, and SBP; Adjust III model adjusts for adjust II + FPG, HbA1c, HDL-C, TG, and TC. BRI, body roundness index.

**Table 3:** Threshold effect analysis of BRI for incident T2DM in men in the NAGALA study.

Inflection point	Hazard ratio (95% CI)	*p* value
Two-piecewise linear regression model		
BRI *Z* score <0.12 (BRI <2.85)	0.70 (0.44, 1.10)	0.1233
BRI *Z* score ≥0.12 (BRI ≥2.85)	1.46 (1.27, 1.67)	<0.001
Log-likelihood-ratio test	0.006	

*Note*. Adjusted for age, alcohol intake, smoking status, fatty liver, SBP, FPG, HbA1c, HDL-C, TG, and TC. BRI, body roundness index.

**Table 4:** ROC analysis for BRI in predicting type 2 diabetes in the NAGALA study.

Variables	AUC (95% CI)	*p* value	Cutoff	Sensitivity (%)	Specificity (%)
Men					
BRI	0.715 (0.684, 0.746)		3.29	61.2	73.2
BMI	0.684 (0.651, 0.717	<0.001	25.05	48.9	78.9
WC	0.700 (0.666, 0.733)	0.007	84.65	60.1	73.7

Women					
BRI	0.758 (0.703, 0.814)		3.05	65.5	75.3
BMI	0.757 (0.704, 0.811)	0.966	21.23	80.5	60.6
WC	0.733 (0.676, 0.789)	0.015	73.45	73.6	63.1

*Note*. *p* value was calculated by comparing with the AUC of BRI. BRI, body roundness index; BMI, body mass index; WC, waist circumference (cm); AUC, area under the curve.

## Data Availability

Data can be downloaded from “DATADRYAD” database (http://www.Datadryad.org).

## Supplementary Materials

Supplementary Materials Supplementary Table 1: baseline characteristics of the participants by sex.
