# Supplementary material for: Relationship between Body Roundness Index and Risk of Type 2 Diabetes in Japanese Men and Women: A Reanalysis of a Cohort Study
Source: Int J Endocrinol. 2021 Dec 29;2021:4535983. doi: 10.1155/2021/4535983 (PMC8731295; doi:10.1155/2021/4535983)
Supplement: Supplementary Materials — Supplementary Table 1: baseline characteristics of the participants by sex. [file 4535983.f1.docx]

**Supplementary Table 1** Baseline characteristics of the participants by sex

|  | | all participants | | |  | participants with T2DM | | |
| --- | --- | --- | --- | --- | --- | --- | --- | --- |
|  |  | Women | Men | *p*-value |  | Women | Men | *p*-value |
|  | participants (n) | 7034 | 8430 |  |  | 87 | 286 |  |
|  | Age (years) | 43.2 ± 8.8 | 44.1 ± 9.0 | <0.001 |  | 47.6 ± 8.5 | 47.0 ± 8.5 | 0.51 |
|  | current smokers | 454 (6.45%) | 3027 (35.91%) | <0.001 |  | 12 (13.79%) | 139 (48.60%) | <0.001 |
|  | Alcohol intake (g/wk) | 1 (0-5.1) | 22 (1-126) | <0.001 |  | 1 (0-1) | 22 (1-120) | <0.001 |
|  | Fatty liver | 486 (6.91%) | 2255 (26.75%) | <0.001 |  | 41 (47.13%) | 182 (63.64%) | 0.006 |
|  | physical activity | 1109 (15.77%) | 1600 (18.98%) | <0.001 |  | 12 (13.79%) | 39(13.64%) | 0.97 |
|  | BMI (kg/m^2^) | 21.0 ± 2.9 | 23.0 ± 3.0 | <0.001 |  | 24.5 ± 4.4 | 25.2± 3.6 | 0.033 |
|  | WC (cm) | 71.7 ± 8.1 | 80.5 ± 7.9 | <0.001 |  | 80.4± 11.8 | 86.5 ± 9.2 | <0.001 |
|  | BRI | 2.57 ± 0.93 | 2.88 ± 0.84 | <0.001 |  | 3.78 ± 1.59 | 3.58 ± 1.01 | 0.769 |
|  | SBP (mmHg) | 109 ± 14 | 119 ± 14 | <0.001 |  | 117 ± 15 | 124 ± 15 | <0.001 |
|  | DBP (mmHg) | 68 ± 10 | 75 ± 10 | <0.001 |  | 73 ± 9 | 78 ± 10 | <0.001 |
|  | TC (mmol/L) | 5.09 ± 0.88 | 5.16 ± 0.85 | <0.001 |  | 5.55 ± 0.93 | 5.39 ± 0.89 | 0.178 |
|  | HDL-C (mmol/L) | 1.65 ± 0.38 | 1.30 ± 0.35 | <0.001 |  | 1.39 ± 0.34 | 1.12 ± 0.30 | <0.001 |
|  | TG (mmol/L) | 0.56 (0.41-0.81) | 0.93 (0.63-1.39) | <0.001 |  | 0.96 (0.73-1.30) | 1.42 (0.91-2.13) | <0.001 |
|  | FPG (mmol/L) | 4.99 ± 0.39 | 5.31 ± 0.37 | <0.001 |  | 5.47 ± 0.41 | 5.66 ± 0.33 | <0.001 |
|  | HbA1c (%) | 5.2 ± 0.3 | 5.2 ± 0.3 | <0.001 |  | 5.6 ± 0.4 | 5.5 ± 0.4 | 0.132 |

**Notes:** Data are presented as mean ± SD, median (Q1-Q3), or N (%).

**Abbreviations:** T2DM, type 2 diabetes mellitus; BRI, body roundness index; BMI, body mass index; WC, Waist circumference; SBP, systolic blood pressure; DBP, diastolic blood pressure; TC, Total cholesterol; HDL-C, HDL-cholesterol; TG, Triglycerides; FPG, Fasting plasma glucose; HbA1c, glycated hemoglobin.
